# Exploring Pharmacist Roles in Telepharmacy for Chronic Disease Management in New York State: A Qualitative Inquiry into Improving Implementation, Patient Communication, and Healthcare Technology Support

**DOI:** 10.7759/cureus.62982

**Published:** 2024-06-23

**Authors:** Azhar Hussain, Alyncia M Bowen

**Affiliations:** 1 Pharmaceutical and Biomedical Sciences, Touro University College of Pharmacy, New York, USA; 2 Healthcare Administration, Franklin University, Columbus, USA

**Keywords:** chronic disease, social determinants of health, health disparities, pharmacists, telepharmacy

## Abstract

Background

This qualitative study aimed to collect information regarding pharmacists' roles in telepharmacy and chronic disease management (CDM). The literature review was conducted on historical overview, CDM overview, social determinants of health (SDOH), disparities, hospital readmissions, adverse drug events, best practices, and global implications of telepharmacy.

Materials and methods

Eleven licensed pharmacists from New York working in retail or hospital settings were interviewed using 16 questions. The interview covered topics such as CDM, hospital readmissions, adverse drug events, best practices, SDOH, and health disparities. The recordings of these interviews were transcribed and coded for each open-ended question, resulting in 136 different codes.

Results

According to the comprehensive review of interview transcripts, there is still an urgent need for communication between healthcare providers and patients, pharmacist training for telepharmacy services and SDOH, and healthcare technology support. CDM can be advanced by pharmacists by understanding patient barriers, SDOHs, health disparities, and pill burdens, as well as incorporating a multidisciplinary approach to patient care. Pharmacists must facilitate open communication, overcome technological barriers, seek support from stakeholders for telepharmacy training, and integrate new mobile applications to improve telepharmacy implementation and operations for providing interventions.

Conclusion

The research provides insights into the comprehensive impact of telepharmacy on healthcare delivery and its potential to transform CDM. With the expansion of telepharmacy, individuals living with chronic diseases can experience improved medication adherence and overall health outcomes.

## Introduction

Pharmacists are an integral part of the healthcare continuum and make up a significant part of the workforce. According to the Bureau of Labor Statistics (BLS), there are 21.7 million workers in the healthcare industry. Of this, 325,480 are pharmacists, making up approximately 1.5% of the workforce [1]. Pharmacists can work in different settings, including retail, hospitals, and large pharmaceutical companies, each with distinct responsibilities. In retail, they interact directly with patients, providing counseling on medication use and answering questions, while in hospitals, they collaborate with healthcare teams to ensure appropriate medication therapy and dosing. Large pharmaceutical companies focus on research and drug development, regulatory affairs, or quality control, focusing more on the manufacturing and distribution aspects of medications [2]. Recognizing the crucial role of pharmacists in managing diseases highlights the need for policies that allow them to take on more responsibilities, including prescribing medications and managing therapies. This is especially important due to the high prevalence of chronic diseases, which require ongoing medical care, and emphasizes the importance of pharmacist intervention.

Chronic diseases are "conditions that last one year or more and require ongoing medical attention or limit activities of daily living or both" [3]. Chronic diseases include but are not limited to heart disease, diabetes, and cancer. In addition to chronic disease management (CDM), telepharmacy allows pharmacists to extend their reach beyond the confines of traditional brick-and-mortar pharmacies. Telepharmacy enables pharmacists to provide medication therapy management (MTM) services, medication counseling, and adherence support to patients in remote areas or those facing challenges accessing in-person pharmacy services. This platform allows pharmacists to interact with patients in real time, addressing their concerns, providing education, and ensuring the safe and effective use of medication. Ultimately, this contributes to improved patient outcomes and overall healthcare accessibility. During the peak of the COVID-19 pandemic, healthcare systems were overwhelmed globally [1,2]. In response, telepharmacy emerged as a critical tool in reaching patients and alleviating strain on the United States' healthcare infrastructure. When providing services to patients through telecommunications, it is essential to consider regulatory gaps and operational barriers. The challenges persist, whether these services are integrated into existing pharmacy structures or new ones. According to the National Association of Boards of Pharmacy, telepharmacy is defined as the "provision of pharmacist care by registered pharmacies and pharmacists through the use of telecommunications to patients located at a distance" [4]. Communities in pharmacy deserts have utilized telepharmacy services to increase access to pharmacists even before the COVID-19 pandemic [5]. Generally, a pharmacy desert is characterized by the lack of pharmacy services in a particular community within a mile radius [6]. Other people use the services to reduce hospital adverse drug events [5]. Adverse drug events are defined as "harmful or unpleasant reactions resulting from an intervention related to the use of medicinal products" that result in negative outcomes [7].

The integration of telepharmacy in CDM is increasing, but as the adoption of telepharmacy is relatively new, the understanding of the pharmacist's perspective regarding its efficacy, challenges, and impact on patient care is limited [6,7]. The objective of this study is to fill the knowledge gap by investigating the experiences, perceptions, and challenges faced by pharmacists when utilizing telepharmacy for CDM. This qualitative study aims to gather information from pharmacists about their roles in telepharmacy and CDM of their patients. The study also aims to gain a deeper understanding of how social determinants of health (SDOH), health disparities, and adverse events affect CDM and to identify best practices for pharmacists to use telepharmacy. These topics are relevant to current market trends, policies, regulations, healthcare costs, and economics.

Current trends and market influences

Telepharmacy markets are competitive and have growth potential. Several independent research organizations have been examining markets and trends for many years. Among these companies are Straits Research, Transparency Market Research, Market Research Future, and Data Bridge Market Research. These research companies have projected significant growth in the electronic health market in the United States in the coming years. In 2021, Straits Research estimated the value of the electronic health market in the United States at approximately $98 million and projected it to grow to $338.15 billion by 2030 [8,9].

Retail giants such as Amazon are joining the field with PillPack Inc., which offers 24-hour access to pharmacists and discounts on medications [10,11]. At the same time, the CDM market, which is expected to reach $20.64 billion by 2029, is being driven by the increasing prevalence of chronic diseases, particularly among the elderly, and the growing demand for digital treatment solutions accelerated by COVID-19 [12]. This market includes a range of technologies and services designed to help individuals manage their conditions effectively, reduce healthcare costs, and improve their quality of life. The merging of telepharmacy and CDM markets indicates significant changes in healthcare delivery, with stakeholders investing to enhance provision and drive efficiency. This trend holds the promise of positive economic outcomes and is reshaping the roles of pharmacists in the competitive healthcare industry [10,13].

Healthcare cost

An estimated 50% of American adults suffer from at least one chronic disease [14]. Considering the increasing prevalence of chronic diseases, it is necessary to develop interventions that increase positive patient outcomes and reduce costs [14]. The integration of telepharmacy into the healthcare system has many advantages. The benefits of doing so include a reduction in the cost of providing healthcare to the public [15]. Therefore, it is likely to impact the economy over the long term significantly. Medication-related incidents per hospital contribute to $261,109 in savings [14,16]. The implementation of telepharmacy leads to a reduction in the number of adverse drug events due to high-risk medications [16].

Similarly, Garrelts et al. found that more interventions resulted in $1,132,144 in savings [17]. Garrelts et al. estimated that the cost savings could be as high as $6,000 if adverse drug events are prevented, errors are caught early, and hospitalizations are reduced [17]. A telepharmacy model can significantly reduce hospital costs and improve healthcare economics in the long run. This has both short- and long-term benefits for the healthcare industry.

Healthcare economics

The United States allocates approximately 90% of its $4.1 trillion healthcare expenditure to address mental disorders and chronic illnesses, with a projected annual increase of 5.1% in government healthcare spending [18]. However, amidst the COVID-19 pandemic, there was a significant 46% decline in government healthcare spending. In light of this, it is imperative to safeguard healthcare funding and direct it toward sustainable endeavors such as telepharmacy. Health spending is influenced by demographics, economic conditions, and specific health-related issues such as chronic conditions. Despite an estimated 4.7%-5.1% increase in prescription drug costs in 2021 due to Medicaid spending, a 4.3% decline was anticipated in 2022. However, a subsequent 5.2% increase in healthcare expenditures is expected over the next five years, largely due to new pharmaceutical programs [18,19].

The integration of telepharmacy into care delivery has the potential to mitigate adverse drug events (ADEs), which contribute to an estimated $30 billion loss in the healthcare industry. ADEs, the third leading cause of clinic and emergency department visits, affect seven million patients annually, resulting in an approximate annual cost of $300 billion to Americans, representing a significant increase from the $145 billion reported in the early 2000s [19]. Notably, medical errors contribute to 22% of readmissions and 41.3% of hospital admissions, amounting to approximately $21 billion in expenditures. The implementation of telepharmacy can reduce the economic burden substantially. Notably, in large healthcare systems, the integration of telepharmacy has demonstrated annual savings exceeding $1,132,144 [20]. Considering the economic implications of telepharmacy, it becomes imperative to identify and address existing gaps.

Policy regulations

Before the onset of the COVID-19 pandemic, the utilization of telehealth services was primarily confined to remote rural areas with specific eligibility prerequisites. However, in reaction to the pandemic, governmental entities such as the United States Health and Human Services (USHHS), the Center for Medicare and Medicaid Services (CMS), and the Joint Commission facilitated the streamlined implementation and operation of telepharmacy services. Policy adjustments encompassed the enactment of the Coronavirus Preparedness and Response Supplemental Appropriations Act of 2020, the accreditation for pharmacists to invoice virtual consultations for COVID-19 diagnosis and treatment, and the relaxation of Health Insurance Portability and Accountability Act (HIPAA) regulations pertaining to pharmacist-patient interactions [21]. Accrediting bodies expedited the endorsement processes for telepharmacy services, rendering them more readily accessible and advantageous for patient care.

In contrast, contemporary health policy prioritizes the delivery of high-quality care using incentive structures, most notably value-based purchasing, necessitating that hospitals fulfill specific benchmarks pertaining to health outcomes and cost containment. It is proposed that the involvement of pharmacists in enhancing patient outcomes could be similarly incentivized, akin to the penalties imposed on hospitals for readmissions that could have been prevented [22]. Notably, in countries such as the United Kingdom, the existing recommendations advocate for the inclusion of pharmacists in policy formation, funding allocation, and the facilitation of vaccination programs. In the wake of the COVID-19 pandemic, comparable revisions have been instituted within the United Kingdom to augment the participation of pharmacists in healthcare decision-making [23].

## Materials and methods

Study design

This qualitative study aimed to explore pharmacists' roles in CDM and to enhance the implementation and operation of telepharmacy. The study utilized a conceptual framework by Sankaranarayanan et al. [24], consisting of three levels: individual, system, and societal impacts, as identified in Table 1. Interviews were conducted to investigate pharmacists' perspectives across these levels.

**Table 1:** Pharmacist interview questions IQ: Interview question.

Level 1: Individual Impacts	
Chronic disease management	IQ1: What steps should be taken to address chronic disease management with your patients?
	IQ2: What challenges do you come across when interacting with your patient's physician regarding concerns?
	IQ3: What barriers have your patients expressed in their care and medication to manage their chronic disease?
	IQ4: How can you monitor your patient's medications and make necessary changes promptly?
Level 2: System Impacts	
Readmissions and adverse drug events	IQ5: Do you use deprescribing methods, and if so, what benefits have you seen in positive patient outcomes? If not, why are deprescribing methods not being used in your practice?
	IQ6: What daily steps do you take to ensure the quality and safety of medication dispensing?
	IQ7: How do you address patient concerns and expedite care to avoid hospitalization?
Best practices	IQ8: What strategic approach have you incorporated to conduct your responsibilities as a pharmacist?
	IQ9: In what ways have you prepared to tackle changes to your job responsibilities by incorporating telepharmacy?
	IQ10: What support have you received from your workplace regarding training in telepharmacy practice?
Level 3: Societal Impacts	
Social determinants of health	IQ11: In what ways do you use telepharmacy to communicate with your patient's providers to integrate a multidisciplinary approach to their care?
	IQ12: What approach do you take to provide your patients with culturally competent education regarding their conditions and medication regimen?
	IQ13: Do you screen for social determinants of health? If so, in what ways have you seen positive outcomes in their health? If not, why are social determinants not accounted for?
Health disparities	IQ14: How have health disparities impacted how you do your work?
	IQ15: In what ways do you feel you lack the necessary skills to address health disparities in the community you serve?
	IQ16: How have you been able to identify disparities present in the community you serve?

Research questions

The study addressed two primary research questions: (1) How can pharmacists contribute to the advancement of CDM? (2) What improvements are needed in telepharmacy implementation and operations for pharmacists to enhance their utilization?

Sampling

Participants were recruited using nonprobability convenience sampling, targeting licensed pharmacists practicing in New York State. A total of 11 pharmacists were recruited, ensuring representation from various practice settings and specialties.

Data collection

Semi-structured interviews were conducted via Zoom, lasting approximately 45-60 minutes each. A set of 16 open-ended questions, grouped into individual, system, and societal impacts, guided the interviews. Audio and video recordings were transcribed manually for analysis.

Data analysis

Thematic analysis was employed to identify patterns and themes in participants' responses. Transcripts were coded using ATLAS.ti software to extract key concepts related to CDM and telepharmacy. The analysis focused on understanding challenges, strategies, and recommendations provided by pharmacists.

Participant demographics

A total of 11 pharmacists participated, consisting of five men (45%) and six women (55%) in the following specialties: critical care, ambulatory care, infectious disease, pharmacotherapy, geriatric, etc. Three (27%) of the participants provided services in retail, whereas the remaining eight (73%) worked in a hospital setting. Table 2 provides a detailed overview of the participants. The participants were labeled Pharmacist 1 (P1) to Pharmacist 11 (P11).

**Table 2:** Participant demographics The "P" in P1 to P11 stands for "Pharmacist".

Participants	Gender	Specialty	Setting
P1	Male	Critical care pharmacist	Hospital
P2	Male	Infectious disease clinical pharmacist	Hospital
P3	Female	Transplant clinical pharmacist	Hospital
P4	Female	Registered pharmacist	Retail
P5	Female	Ambulatory care clinical pharmacists	Hospital
P6	Female	Ambulatory care clinical pharmacists	Hospital
P7	Male	Ambulatory care clinical pharmacists	Hospital
P8	Female	Pharmacotherapy specialists	Retail
P9	Female	Ambulatory care clinical pharmacists	Hospital
P10	Male	Registered pharmacist	Retail
P11	Male	Geriatric pharmacy specialty certification	Hospital

Data collection instrument

The interview questions, categorized by theme, covered topics such as CDM, readmissions and ADEs, best practices, SDOH, and health disparities. Table 3 presents a comprehensive list of interview questions and corresponding codes.

**Table 3:** Level 1: individual (chronic disease management) interview questions 1–4, with codes and response percentages IQ: Interview question; N: Number of responses; CDM: Chronic disease management; CMRs: Comprehensive medication reviews. IQ1 N = 29. IQ2 N = 22. IQ3 N = 46. IQ4 N = 20.

IQ1 Codes		IQ2 Codes		IQ3 Codes		IQ4 Codes	
Evaluating patients' understanding of their disease	21%	Communication/access to physicians/providers outside the hospital clinic	36%	Keeping themselves motivated/inspired to take medication	15%	Reviewing medication chart/pill box on clinic visits	15%
Building trust with patients	17%	Compliance in handling medication	9%	Pill burden/difficulty in understanding medication	15%	Efficient use of telehealth/telemedicine	15%
Familiarity with prevalent chronic disease	10%	Creating trust in pharmacists to manage medication	9%	Lack of health literacy	13%	Regular follow-up with patients	10%
Awareness about updated clinical guidelines/compliance	10%	Don't consider pharmacists important in patient management	9%	Appropriate language to converse with patients	11%	Checking patients’ vitals	10%
Understanding patient barriers	10%	Patient's not getting prior authorization from physicians	9%	Lack of financial ability	11%	Review patient profile/CMRs	10%
Help understanding medication	10%	Lack of pharmacist within specialty team to make recommendations	9%	Issues with insurance coverage	9%	Developing personal relationships with patients	10%
Understanding health disparities	7%	Dealing with patients without insurance	9%	Transportation issues	7%	Electronic tracking of medication	10%
Managing diagnosis aspects	7%	Problems in using online tools to update medication	5%	Difficulty in changing lifestyles/food habits	7%	Identifying laboratory abnormalities/side effects of medication	5%
Access to physician	3%	Understanding the importance of chronic disease management in preventing disease progression	5%	A high number of health disparities	7%	Prior authorization and meeting with the doctor	5%
Prior authorization	3%			Difference in recommendation of pharmacist and physician	2%	Encouraging teaching to use pill box	5%
Verifying CDM need	3%			Ease of access to providers	2%	Collaborative Drug Therapy Management (CDTM)	5%
				Electronic literacy/not able to use telehealth	2%		

Ethical considerations

Approval was obtained from the Institutional Review Board of Franklin University before data collection (approval number: IRB-2023-39). Participants provided informed consent and were assured of confidentiality and data security throughout the study.

## Results

Individual impact: chronic disease management

The individual-level theme impacts included the role that pharmacists play in CDM. As shown in Table 3, there were 43 unique codes resulting from these four questions, with 117 responses from pharmacists. The 117 responses were received from pharmacists who identified multiple codes for a single question. The results are summarized in Table 3, along with the total number of responses per interview question. For interview question (IQ) 2, P5 identified three codes: communication and access to providers, not considering pharmacists’ critical in-patient management, and creating trust in pharmacists for managing medications. According to P5, pharmacists are not accepted as part of the provider team in New York in contrast to their experiences in Florida, where pharmacists are accepted as members of the provider team. P5 stated, “New York City area is a little bit behind the times, I would say a little slower to accept and embrace the pharmacist as part of the health care team.” In IQ2, 36% (8/22) of responses from pharmacists identified that communication or access to providers outside of hospital settings was challenging when interacting with their patients. Furthermore, the top two codes for IQ1 were evaluating patients' understanding of their disease (21% or 6/29) and building trust with patients (17% or 5/29), both of which are critical steps in addressing CDM with patients. With 46 responses, IQ3 identified the most significant barriers experienced by patients in managing chronic conditions: maintaining motivation to take medication (15% or 7/46), pill burden (15% or 7/46), and lack of health literacy (13% or 6/13). Lastly, when examining IQ4, pharmacists identified two of the most effective methods for monitoring their patients’ medications, such as reviewing medications in charts/pillboxes during visits and using telehealth and telemedicine efficiently (15% or 3/20).

System impact: readmissions, adverse drug events, and best practices

The system-level theme impacts the included pharmacists' role in hospital readmissions, ADEs, and best practices. Tables 4, 5 demonstrate that these IQs led to 133 responses with 49 distinct codes. The questions at this level received the highest number of responses. There is a possibility that this is because 73% of participants were from a hospital setting where adverse events, readmissions, and best practices are readily applied. IQ5 received the greatest number of responses. About 48% (10/21) of the responses identified that reducing pill burden was one of the benefits of positive patient outcomes in deprescribing methods. Six responses out of 21 (29%) indicated that monitoring patients' medication and making changes as soon as possible can help them manage adverse events and side effects. IQ6 pertains to pharmacists assuring accurate medication dispensing (28% or 8/29), tracking the medication history of a patient (24% or 7/29), and preventing drug interactions (17% or 5/29). As part of IQ7, eight of the 25 total responses (32%) indicated that pharmacists agreed that open communication with their patients and regular follow-up are the best strategies for addressing their concerns and expediting care to reduce hospitalization. In addition, 20% (5/25) of responses indicated that they provided patients with the required medications, and 16% (4/25) said they counseled patients to understand their medications. In IQ8, the top four codes included the following: regularly following up with patients to ensure compliance (4/23 or 17%), setting protocols to ensure that the patient is taking the correct medication (4/23 or 17%), counseling the patient (3/23 or 13%), and being accessible to the patients (3/23 or 13%), which were the methods used to monitor patients’ medications. For IQ9, pharmacists were asked how they would change their job responsibilities to accommodate telepharmacy. About 38% (6/16) of the responses indicated that understanding and overcoming technological difficulties would be the most appropriate method. In addition, the top responses for IQ10 included that pharmacists did not receive much support (32% or 6/19) and were not provided an overview of the technology from the workplace regarding telepharmacy training (26% or 5/19).

**Table 4:** Level 2: system (readmission/adverse drug events) interview questions 5–7, with codes and response percentages IQ: Interview question; N: Number of responses. IQ5 N = 21. IQ6 N = 29. IQ7 N = 25.

IQ5		IQ6		IQ7	
Reduce pill burden/as per patient needs	48%	Ensuring the dispensing of medication goes appropriately	28%	Open communication/regular follow-up with patients	32%
Help patients with adverse actions/side effects	29%	Keeping track of patients’ medication regime/history	24%	Facilitate patients with required medications	20%
Reduce prescribing based on duplication	10%	Prevent drug interaction	17%	Counseling patients to understand medication	16%
Help to motivate their gut	5%	Assist with prior authorization	7%	Escalate concerns with the provider if needed	16%
Help patients understand the medications	5%	Clear/easy-to-read directions on prescription	7%	Keeping track of supply	8%
Do comprehensive medication reconciliation	5%	E-prescribing	7%	Help patients manage pill box	4%
		Barcode scanning for correct medication	3%	Delivering medicines/same-day delivery to patients	4%
		Proper storage of medicines	3%		
		Refer to nursing/administration notes	3%		

**Table 5:** Level 2: system (best practices) interview questions 8–10, with codes and response percentages IQ: Interview question; N: Number of responses; CDM: Chronic disease management. IQ8 N = 23. IQ9 N = 16. IQ10 N = 19.

IQ8		IQ9		IQ10	
Regular follow-up with patients on compliance	17%	Understanding/overcoming technological difficulties	38%	Did not receive much support	32%
Setting protocols to ensure the patient is on the correct medication	17%	Flexible communication options	25%	Technology overview/support	26%
Patient counseling/help patients understand medicine	13%	Handling more patients	13%	Provided virtual training	11%
Being accessible to patients	13%	Standardize laboratory measurement procedure	13%	Training modules to be completed	11%
Inventory management	4%	Multitasking	6%	Handling phone calls	5%
Flexible home delivery options for patients	4%	Understanding billing process	6%	On-the-job training	5%
Centralized database to track due dates	4%			Training on CDM	5%
Review notes from providers	4%			Ambulatory care training by experts	5%
Addressing patient issues faster	4%				
Checklist of patient requirements	4%				
Clear labeling on pill bottles	4%				
Participate in teaching opportunities	4%				
Continued education	4%				

Societal impact: SDOH and health disparities

The societal level impacts included SDOH and health disparities. Among the 110 responses, the researcher found 43 distinct codes for these IQs, as shown in Tables 6, 7. The lowest number of responses was received for this level of questions. A possible explanation for this may be that the majority of the participants came from a hospital setting where social workers rather than pharmacists examine SDOH and disparities. The greatest number of responses were received from IQ16, for which 47% (8/17) of responses reported that patient interviews and building relationships were the primary ways by which pharmacists identified disparities. As a means of interacting with patients’ physicians, 40% (6/15) of responses were identified using the telephone in IQ11. As identified in IQ12, pharmacists can provide culturally competent education to their patients in seven different ways. The most used approaches are translation services (22% or 5/23), patient education (22% or 5/23), understanding food culture (17% or 4/23), and understanding cultural disparities (17% or 4/23). The pharmacists in IQ13 described how they consider SDOH when assessing positive health outcomes for patients. This includes the provision of health insurance (24% or 5/21), the encouragement of social support (19% or 4/21), and the improvement of economic conditions (19% or 4/21). In IQ14, the pharmacists identified how disparities affect their work. Understanding socioeconomic status (39% or 7/18) and educating patients about their medications (17% or 3/18) were the main ways by which disparities affected the work of pharmacists. In IQ15, the pharmacists identified eight areas in which they lack the skills to address disparities, including understanding patient resources (31% or 5/16), addressing social disparities (25% or 4/16), and working with people with low health literacy (13% or 2/16).

**Table 6:** Level 3: society (social determinants of health) interview questions 11–13, with codes and response percentages IQ: Interview question; N: Number of responses. IQ11 N = 15. IQ12 N = 23. IQ13 N = 21.

IQ11		IQ12		IQ13	
Communicate via phone	40%	Translation services/interaction in the primary language	22%	Insurance coverage	24%
Use of electronic health records	27%	Education patients about medication	22%	Encourage them to find social support	19%
Text/chat services	13%	Understanding food culture	17%	Economic situation	19%
Establishing collaborative practices	13%	Understanding cultural disparity	17%	Educating patients about medication	10%
Communicate by fax	7%	Building connections with patients	13%	Understand goals/build a relationship with the patient	10%
		Emergency contact information	4%	Already screened by the primary provider	5%
		Standardized pamphlets with instructions	4%	Don’t screen/unsure of the process	5%
				Transportation barriers	5%
				Help make accessible changes	5%

**Table 7:** Level 3: society (health disparities) interview questions 14–16, with codes and response percentages IQ: Interview question; N: Number of responses. IQ14 N = 18. IQ15 N = 16. IQ16 N = 17.

IQ14		IQ15		IQ16	
Understanding socioeconomic status	39%	Understanding the right resources for patients	31%	Patient interviews/building relationships	47%
Educating patients about medication	17%	Addressing social disparities	25%	Economic status	18%
Providing resources to support	11%	Dealing with low health literacy	13%	Understanding of health literacy	12%
Looking for charitable medication options	11%	Educating yourself by practicing in different areas	6%	Understanding communities/speaking to community leaders	6%
Helping patients with appointments/medications	11%	Creating reliable communication channels	6%	Type of medical insurance	6%
Translation services/interaction in the primary language	6%	Language barrier	6%	Reliable phone services	6%
Electronic literacy/not able to use telehealth	6%	Administration technique	6%	Institutional research	6%
		Handling insurance issues	6%		

## Discussion

Chronic disease management

Medication management plays a critical role in the pharmacist's responsibilities within CDM. CDM encompasses an interdisciplinary approach to healthcare, encompassing self-management, provider-patient relationships, prevention, evidence-based guidelines, health evaluation, outcome assessment, and enhancement of quality of life [25]. Healthcare teams are encouraged to implement population-specific programs, evidence-based guidelines, collaboration, risk identification, and monitoring of patient progress [25]. An important consideration in the development of disease management programs for specific populations is the influence of socioeconomic status [14,25].

Pharmacists should prioritize targeted therapies for high-risk patients, closely monitor prescribing patterns, educate physicians regarding new treatments, and carefully track medication management expenditures to establish sustainable CDM practices. In instances of consistent medication misuse or abuse, pharmacists should both educate the patient and consult with the patient's physician. Many of these activities can be effectively facilitated through telepharmacy.

Patients presenting with chronic conditions necessitate comprehensive management from an interdisciplinary care team. An estimated 56% of individuals with chronic diseases are concurrently prescribed a minimum of five medications [26], leading to polypharmacy [27]. This, in turn, has the potential to diminish medication adherence, with only 50% of prescribed medications being consumed as directed [26]. Telepharmacy serves as a viable solution for improving medication safety and ameliorating adherence and treatment gaps within this patient population [16,26]. Inadequate medication adherence presents a substantial challenge to the healthcare system. Policymakers are urged to develop effective strategies to enhance adherence [28]. Telepharmacy empowers pharmacists to oversee adherence data, thereby guaranteeing that patients adhere to their prescribed medication regimens. This technology can effectively mitigate shortcomings in medication adherence by granting patients improved access to pharmacists over an extended period.

Establishing a robust patient-pharmacist rapport is imperative. Employing multiple communication touchpoints, such as phone calls and emails, is associated with an increased likelihood of patients adhering to their treatment regimens, thereby amplifying engagement and satisfaction [6]. The cultivation of trust between patients and pharmacists facilitates the disclosure of lifestyle, financial barriers, and gaps in health management, which is pivotal for effective medication adherence [6,28].

Pharmacists engage with patients frequently, often surpassing the frequency of interactions with physicians. This allows pharmacists to promptly provide essential medication information. They encounter patients 1.5 to 10 times more frequently than physicians, which provides opportunities for patient education, medication counseling, and management, leading to increased medication adherence and averting therapy duplication [29]. Patients often exhibit greater ease in discussing medication concerns with pharmacists, who can consequently aid in comprehending physician diagnoses and treatment plans [30]. Despite the pivotal role played by pharmacists, communication barriers with physicians persist. Telepharmacy seeks to address these gaps, thereby reducing ADEs and hospitalizations, ultimately leading to a decrease in healthcare costs [31].

In summary, pharmacists play a vital role in the management of chronic diseases through the provision of crucial medication management, patient education, and adherence support. Telepharmacy serves to augment these capacities, guaranteeing ongoing patient assistance and enhanced health outcomes. This interdisciplinary method underscores the necessity of integrating pharmacists into CDM to effectively bolster patient quality of life.

Readmission, adverse drug events, and best practices

Telepharmacy plays a critical role in preventing ADEs and significantly reducing healthcare costs. ADEs are classified into six types: dose-related, non-dose-related, dose and time-related, time-related, withdrawals, and failed therapy [7]. Each year, medical errors result in 7,000 deaths and 250,000 injuries, costing over $2 billion in the United States alone [32]. The introduction of telepharmacy in hospitals has been proven effective in reducing medical errors and hospitalizations [32,33].

Pharmacists play a crucial role in the healthcare system by implementing strategic approaches such as regular patient follow-ups, clear labeling, and deprescribing to manage polypharmacy and minimize ADEs. These practices help address patient concerns about medication burden, improve understanding, and ultimately reduce hospital readmissions and extended stays [27]. Notably, 48% of pharmacists use deprescribing to reduce pill burden, and 10% use it to eliminate medication duplication, highlighting the necessity of a multidisciplinary approach to healthcare [34]. The effectiveness of pharmacist-led teams in reducing readmission rates, compared to physician-only teams, is well-documented. These teams correct drug management errors, detect duplications, ensure proper dosages, and significantly lower occurrences of ADEs [34]. Additionally, telepharmacy initiatives have demonstrated cost savings of over $700,000 annually by managing medical errors that result in adverse events [32].

The healthcare industry encourages telepharmacy as part of routine care, following standardized best practices supported by organizations such as the American Society of Health-System Pharmacists (ASHP) and CMS. It involves pharmacist training, secure patient communications, and detailed documentation of patient interactions [35,36]. Additionally, incorporating screenings for SDOH into pharmacist responsibilities ensures a comprehensive approach to patient care [37]. Despite some patients' technological challenges, telepharmacy has been proven to improve patient retention and follow-up care. However, for its broader implementation, specialized technology and comprehensive training in telepharmacy are crucial [38].

In conclusion, telepharmacy, supported by best practices and strategic pharmacist interventions, offers a promising solution to reduce ADEs, lower healthcare costs, and improve patient outcomes. Continued emphasis on multidisciplinary approaches and deprescribing can further enhance its effectiveness in managing chronic diseases and polypharmacy.

Social determinants of health and health disparities

SDOH refers to the conditions where people live, learn, work, and play that can impact their health and quality of life. Various factors, such as social support networks and access to medical care, can affect treatment adherence and medication effectiveness. The COVID-19 pandemic heightened these issues. Telepharmacy allows pharmacists to address SDOH in patients by discussing health literacy, housing stability, employment, medication access, insurance, and transportation.

Pharmacists need to recognize their biases when working with marginalized communities to ensure that everyone receives fair and equal care. For example, different racial and ethnic groups may have varying levels of willingness to receive vaccinations [38]. Additionally, people with hearing impairments or those without internet access may encounter difficulties with telepharmacy. The lack of culturally appropriate and bilingual medication information in pharmacies can widen educational gaps [18,20,38]. These gaps can impact CDM by affecting patients' understanding of their medication regimen.

To overcome these barriers, pharmacists need to create an inclusive environment and establish trust with patients, as patients often visit their pharmacists more frequently than their doctors [37]. Effective communication and building strong relationships are crucial, as patients are more likely to openly share social challenges with trusted pharmacists [6,37]. Using translation services, providing culturally sensitive education, and addressing systemic barriers are crucial strategies for improving medication adherence and establishing rapport with the patient [38].

Health disparities refer to the differences in health outcomes linked to social, economic, or environmental disadvantages, and they significantly impact pharmacy practice [6,39]. Pharmacy deserts are often found in minority communities, and they underscore these disparities as residents in these areas face multiple barriers, such as language issues, poverty, and lack of health insurance [6,37]. While telepharmacy can help address some of these issues, challenges such as broadband access and health literacy still exist. Pharmacists need to provide culturally competent care, including offering translation services and patient education, to bridge the gaps in patient understanding and medication adherence [40-42]. It is crucial to develop pharmacists' skills in addressing SDOH and health disparities as many pharmacists lack experience in these areas [37]. Collaborating with social workers and other healthcare professionals can enhance patient support, ensuring comprehensive care [37].

In conclusion, telepharmacy offers a valuable tool for addressing health disparities by enhancing patient-pharmacist communication and providing culturally competent care. Building strong, unbiased relationships with patients and integrating pharmacists into multidisciplinary teams can significantly improve patient outcomes and reduce health disparities.

Research limitation

Convenience sampling may introduce selection bias, and findings may not be generalizable beyond the study population. Additionally, the study's reliance on self-reported data and the potential for social desirability bias could influence participants' responses. Also, the sample size was only a snapshot of pharmacists in New York. As a large city, New York City's pharmacist population may differ greatly from other states. According to the literature, a sample size of 10-15 pharmacists was recommended to reach data saturation, but in our study, the sample size ultimately totaled 11 pharmacists. This lack of variability in the data may lead to bias, resulting in skewed data that limits the understanding of medical practices, demographics, and disease prevalence in the area where pharmacists are practicing.

Another limitation was that we did not define “telepharmacy” when contacting the pharmacists to request their research participation. A few pharmacists thought there was a software application called “Telepharmacy” and refused to participate because they were inexperienced with the software.

Lastly, coding was done twice, once by ATLAS.ti and then again manually. As ATLAS.ti established the codes, any manual entry would have biased them. In contrast to ATLAS.ti, which provided single words, the final codes were phrases that provided a more accurate representation of the responses.

Recommendations for further research

We offer several recommendations to advance the knowledge and improve the quality of patient care at the rapidly evolving intersection of telepharmacy and CDM.

Integrating telepharmacy services with CDM platforms should be the focus of future research. Pharmacists and healthcare providers could communicate in real time, improving medication management and increasing drug adherence for chronically ill patients.

Using user-friendly mobile applications tailored for pharmacists and patients can enhance the impact of telepharmacy on CDM. Through the simplification of the interface, patient access and engagement will be enhanced, ultimately resulting in improved medication adherence and patient outcomes. Healthcare delivery can be revolutionized with these patient-centric applications.

Pharmacists do not currently receive compensation for their time spent on telepharmacy. Policymakers, healthcare systems, and government agencies should collaborate to develop billing codes and incentive programs and discuss fair reimbursement for pharmacists' services.

Future researchers should examine pharmacists' role in addressing health disparities and SDOH in their communities. Additionally, pharmacy schools, industry experts, healthcare systems, and even community pharmacies might need to emphasize education surrounding disparities and SDOH.

These recommendations are based on the findings of this study. In the study, only 5% of the pharmacists received training in CDM and telepharmacy. As a result, these fields must be examined in further depth to determine their full potential. As mentioned in recommendation 2, application improvements are necessary to address gaps found during this research. Several pharmacists noted that culturally appropriate applications were lacking, particularly regarding technology literacy. In approximately 6% of pharmacy practices, pharmacists reported that patients were unable to use teleservices because they lacked electronic literacy. Per recommendation 3, prior authorizations were obstacles to many pharmacists’ responsibilities. Pharmacists do not receive payment for contacting insurance companies to obtain prior authorizations. Doctors are responsible for contacting insurance companies for authorizations. Pharmacists do not have the diagnosis codes necessary to get insurance authorizations. In the interviews, the participants indicated that this barrier existed. Approximately 9% of the participants reported that patients do not obtain prior medication authorization from their physicians. This makes it difficult for pharmacists to interact with providers regarding medication concerns. Furthermore, 7% of the participants assisted with prior authorizations to ensure medication dispensing quality and safety. These efforts to ensure prior authorization have gaps that need to be addressed. Given the importance of prior authorization, pharmacists would be expected to help more frequently. Thus, recommendation 5 is that pharmacists should be appropriately compensated and included in the collaborative effort to discuss the implementation of new policies.

Lastly, according to this study's findings, pharmacists do not feel that they possess the necessary skills to deal with disparities, understand the proper resources for patients, deal with low health literacy, and educate themselves on SDOH. The findings of this study determined the recommendations presented here.

## Conclusions

The data gathered from the interviews provides a valuable perspective on the role of telepharmacy in CDM. This research sheds light on telepharmacy’s multifaceted impact on healthcare delivery and its potential for revolutionizing CDM. One of the key findings of this study is that the burden of chronic disease is increasing every day; therefore, telepharmacy might help to reduce the burden of chronic disease by significantly improving patient access to healthcare. With the expansion of telepharmacy, individuals living with chronic diseases might experience an improvement in medication adherence and overall health outcomes due to pharmacists' counseling and education. Furthermore, the interviews revealed that effective communication and collaboration between healthcare providers are paramount in telepharmacy to ensure comprehensive patient care. As a result of this study, telepharmacy has been demonstrated to be a useful tool for managing chronic diseases. By incorporating a multidisciplinary approach, pharmacists can advance CDM by understanding patient barriers, SDOH, health disparities, and pill burden. Despite these advantages, it is important to recognize the dilemmas and ethical concerns associated with telepharmacy.
